# Supplementary material for: MetaRibo-Seq measures translation in microbiomes
Source: Nat Commun. 2020 Jun 29;11:3268. doi: 10.1038/s41467-020-17081-z (PMC7324362; doi:10.1038/s41467-020-17081-z)
Supplement: Supplementary file 10 — Supplementary Data 7 [file 41467_2020_17081_MOESM10_ESM.zip › File2/Confidence_High_Taxonomy/90945_out.krona.html]

Javascript must be enabled to view this page.

members
magnitude
magnitudeUnassigned
count
unassigned
taxon
rank

90945\_out

2

2
2
superkingdom

1239
2
phylum

2
186801
class

2
186802
order

186803
2
family

genus
2039240
2

160404
2

SRS019601\_contig\_number\_12784SRS048060\_contig\_number\_contig-100\_17004.17005
species
